# Atg3 Overexpression Enhances Bortezomib-Induced Cell Death in SKM-1 Cell

**DOI:** 10.1371/journal.pone.0158761

**Published:** 2016-07-08

**Authors:** Lin Zhuang, Yan Ma, Qian Wang, Jing Zhang, Chen Zhu, Lu Zhang, Xiaoping Xu

**Affiliations:** Department of Hematology, Huashan Hospital, Shanghai Medical College, Fudan University, Shanghai, China; Queen's University Belfast, UNITED KINGDOM

## Abstract

**Background:**

Myelodysplastic syndrome (MDS) is a group of heterogeneous hematopoietic stem cell malignancies with a high risk of transformation into acute myeloid leukemia (AML). Clonal evolutions are significantly associated with transformation to AML. According to a gene expression microarray, *atg3* is downregulated in MDS patients progressing to leukemia, but less is known about the function of Atg3 in the survival and death of MSD/AML cells. Moreover, the role of autophagy as a result of bortezomib treatment is controversial. The current study was designed to investigate the function of Atg3 in SKM-1 cells and to study the effect of Atg3 on cell viability and cell death following bortezomib treatment.

**Methods:**

Four leukemia cell lines (SKM-1, THP-1, NB4 and K562) and two healthy patients’ bone marrow cells were analyzed for Atg3 expression via qRT-PCR and Western blotting analysis. The role of Atg3 in SKM-1 cell survival and cell death was analyzed by CCK-8 assay, trypan blue exclusion assay, DAPI staining and Annexin V/PI dual staining with or without bortezomib treatment. Western blotting analysis was used to detect proteins in autophagic and caspase signaling pathways. Electron microscopy was used to observe ultrastructural changes after Atg3 overexpression.

**Results:**

Downregulation of Atg3 expression was detected in four leukemia cell lines compared with healthy bone marrow cells. Atg3 mRNA was significantly decreased in MDS patients’ bone marrow cells. Overexpression of Atg3 in SKM-1 cells resulted in AKT-mTOR-dependent autophagy, a significant reduction in cell proliferation and increased cell death, which could be overcome by the autophagy inhibitor 3-MA. SKM-1 cells overexpressing Atg3 were hypersensitive to bortezomib treatment at different concentrations via autophagic cell death and enhanced sensitivity to apoptosis in the SKM-1 cell line. Following treatment with 3-MA, the sensitivity of Atg3-overexpressing cells to bortezomib treatment was reduced. Atg3 knockdown blocked cell growth inhibition and cell death induced by bortezomib.

**Conclusion:**

Our preliminary study of Atg3 in the high-risk MDS cell line suggests that Atg3 might be possibly a critical regulator of autophagic cell death and a gene target for therapeutic interventions in MDS.

## Introduction

Myelodysplastic syndrome (MDS) is a group of heterogeneous hematopoietic stem cell malignancies characterized by peripheral blood cytopenias due to ineffective hematopoiesis, bone marrow dysplasia and increased risk of transformation into acute myeloid leukemia (AML) [1]. Many patients suffer from complications related to refractory cytopenias, and approximately one-third of patients with MDS may progress to AML [2]. Once transformed to AML, patients have a poor prognosis and a high risk of death. Recently, many studies have demonstrated that the progression of MDS is caused by the acquisition of cytogenetic abnormalities [3,4]. Our previous findings showed that *atg3* is significantly downregulated in MDS patients with leukemic evolution [5], which confirms that clonal evolution is significantly associated with transformation to AML.

Autophagy is an active homeostatic lysosomal degradation process for the removal or breakdown of cytoplasmic components [6]. Autophagy requires generating double membrane-bound structures termed autophagosomes that are regulated by multiple autophagy-related genes (*atgs*) to recycle intracellular proteins and organelles. There are two ubiquitin-like conjugation processes essential for the formation of autophagosomes during autophagy that attach Atg12 to the target protein Atg5 and microtubule-associated protein light chain 3 (LC3) to the lipid phosphotidylethanolamine (PE) [7]. Atg3, an E2-like molecule, contributes to the conjugation of LC3 to PE. Accumulated evidence has shown that Atg3 is a critical regulator of cell survival and death. Oral et al. confirmed that Atg3 is a direct target of caspase-8 and plays a key role in autophagy regulation during cell death and survival [8]. Another study showed that whether Atg3 triggers autophagy or apoptosis in intestinal epithelial cells depends on the circumstance [9]. Recently, Radoshevich et al. found that Atg3 conjugation to Atg12 affects mitochondrial homeostasis and plays a role in cell death mediated by the mitochondrial pathway [10]. However, the role of Atg3 in the survival and death of MDS/AML cells remains elusive.

The degradation of intracellular proteins is involved in the regulation of various cellular processes, including cell growth, apoptosis, cell cycle, DNA repair and cell differentiation. The two primary protein degradation pathways are the ubiquitin-proteasome system (UPS) and autophagy-lysosome system. Although the two systems are thought to be independent, much evidence shows that crosstalk between the two systems exists. Ubiquitination and proteasome-mediated degradation are two major processes in UPS-mediated proteolysis. Bortezomib is a selective and reversible inhibitor of the 26S proteasome and is widely used for the treatment of multiple myeloma and mantle cell lymphoma. Recent studies have demonstrated that bortezomib can improve the response and survival of MDS patients and shows therapeutic efficacy in patients with high-risk MDS or AML [11–13]. As a potent stress inducer, bortezomib treatment results in autophagy and the accumulation of non-degraded proteins. However, whether bortezomib induces autophagy that is pro-survival or pro-death is controversial. Recently, Fang et al. found that bortezomib induces the autophagy pathway to initiate cell death in MDS/AML [14].

The role of autophagy in cancer cells is complex. Autophagy has been demonstrated as an important pro-survival process for conferring resistance to chemotherapy, radiation therapy and immunotherapy in cancer cells by recycling nutrients and clearing damaged organelles [15]. However, recent studies have shown that defective autophagy can promote the development of MDS and leukemia [16]. Enforced overactivation of autophagy can also lead to cell death. Whether autophagic cell death exists in cancer is still controversial [17,18]. Therefore, efforts to understand and modulate the autophagy pathway will provide new approaches to cancer therapy and prevention.

In the present study, we observed that Atg3 overexpression induced autophagic cell death. Moreover, Atg3 enhanced the chemosensitivity to bortezomib via autophagic cell death and increased apoptosis. Furthermore, the autophagy inhibitor 3-methyladenine (3-MA) could overcome this chemosensitivity. Our findings show that autophagy can act as a tumor suppressing mechanism and enhance the cytotoxicity of bortezomib.

## Materials and Methods

### Cells and cell culture

The MDS cell line SKM-1 was purchased from Health Science Research Resources Bank in Japan. The acute monocytic leukemia cell line THP-1, the acute promyelocytic leukemia cell line NB4, and the chronic myelogenous leukemia cell line K562 were purchased from the Institute of Biochemistry and Cell Biology, Chinese Academy of Sciences (Shanghai, China). The cells were cultured in RPMI-1640 medium (Life Technologies, Gaithersburg, MD, USA) with 10% heat-inactivated fetal bovine serum (Gibco, Invitrogen, Carlsbad, CA, USA) and 1% penicillin/streptomycin (Gibco). The cells were kept in a humidified incubator at 37°C with 5% CO_2_ and 95% air. Bone marrow mononuclear cells of MDS and healthy patients were isolated from volunteer donors, from whom written formed consent for experimental use was obtained. The protocols received approval from the ethics committees of the Institutional Review Board of Huashan Hospital, Fudan university (Permit Number: 2011–037).

### Reagents

Bortezomib was purchased from Xian-Janssen Pharmaceutical Ltd. (Xian, Shanxi, China), and 3-MA was purchased from Sigma-Aldrich. Bortezomib and 3-MA were dissolved in phosphate-buffered saline (PBS) and diluted in PBS to the desired concentration. Bafilomycin A1 (Baf A1) was purchased from Sigma-Aldrich and was diluted to the desired concentrations before use.

### Gene expression analysis by real-time PCR

After cell lysis, total RNA was extracted using the AllPrep DNA/RNA/Protein Mini Kit (Qiagen, Germany), and then the samples were reverse transcribed using the PrimScriptTM RT Reagent Kit (TaKaRa, China) as described in the manufacturer’s protocol. The cDNA was used as a template for qRT-PCR in a Real-Time PCR 7500 System (Applied Biosystems, Invitrogen) with SYBR Premix Ex Taq (TaKaRa, China) in a 40-cycle PCR reaction. The denaturing, annealing, and extension conditions of each PCR cycle were 95°C for 30 s, 95°C for 5 s, and 60°C for 34 s. The gene-specific primer pairs were as follows: Atg3 forward, 5’ACTGATGCTGGCGGTGAAGATG3’; Atg3 reverse, 5’GTGCTCAACTGTTAAA GGCTGCC3’; GAPDH forward, 5’GAAGGTGAAGGTCGGAGTC3’; and GAPDH reverse, 5’GAAGATGGTGATGGGATTTC3’. The relative expression levels were calculated by the 2^-^ΔΔCt method. The mRNA levels of the target gene were normalized to the levels of GAPDH.

### Construction of the lentiviral plasmid

The ATG3 gene overexpression lentivirus was constructed by GeneChem Company (Shanghai, China). Human ATG3 cDNA (NM_022488.3) was amplified by PCR and then inserted into the GV287 lentiviral vector, which was linearized with Age I and BamH I. The correctly constructed plasmid was confirmed by sequencing.

### Lentiviral transduction

Lentiviral particles were produced by co-transfecting lentiviral plasmids into 293T cells with two helper constructs. Titers of 2–5×10^7^ TU/ml were routinely achieved. SKM-1 cells were infected with 20 MOI lentiviral particles with FLAG-tagged ATG3 overexpressing vector and empty vector (VECTOR-GFP). Polybrene, an enhancing reagent, was added at 5 μg/ml to improve the transfection efficiency. After 8–12 h, the medium was exchanged for complete medium. At 72 h after infection, the cells were observed under a fluorescence microscope (Nikon, Tokyo, Japan). Atg3 expression was detected by Western blotting. Cells with stable GFP expression were harvested for further experiments. Each experiment was performed in triplicate.

### Western blotting

Total protein (30 μg) and pre-stained molecular weight markers were separated by SDS-PAGE and electro-transferred onto a polypropylene difluoride membrane (Millipore, Billerica, MA, USA). The membranes were blocked by incubating with Tris-buffered saline containing 0.1% Tween-20 and 5% non-fat milk for 2 h at room temperature. Then, the membranes were probed at 4°C overnight with the following primary antibodies: from Cell signaling (Danvers, MA, USA): rabbit anti-Atg3 (catalog no.:3415; 1:1000 dilution), rabbit anti-LC3B (12741; 1:1000), rabbit anti-GAPDH (5174; 1:1000), rabbit anti-p62 (8025; 1:1000), rabbit anti-p-AKT (S473) (9271; 1:1000), rabbit anti-p-mTOR (S2448) (5536; 1:1000), rabbit anti-caspase-3 (9662; 1:1000) and mouse anti-caspase-8 (9746; 1:1000) antibodies; from Zen BioScience (Chengdu, Sichuan, China): mouse anti-FLAG (390002; 1:2000). After washing, the membrane was incubated with a secondary antibody conjugated to horseradish peroxidase for 1 h at room temperature. Finally, the membrane was prepared with enhanced chemiluminescence substrate (Pierce, Rockford, IL, USA) and visualized on a LAS-3000 Luminescence Image Analyzer (Fujifilm, Tokyo, Japan). All antibodies were purchased from Cell Signaling Tech Inc.

### Immunofluorescence analysis

After 72 h of transfection, cells were seeded onto a cover glass and fixed in 4% formaldehyde for 30 min at room temperature before cell permeabilization with 0.1% Triton X-100. The cells were subsequently saturated with PBS containing 2% bovine serum albumin for 1 h at room temperature. The cells were then incubated with an anti-LC3 antibody (Cell Signaling Technology) followed by Alexa Fluor 555-conjugated immunoglobulin. After a 1-h incubation, the cells were incubated with 0.5 μg/ml 4,6-diamidino-2-pheny-lindole (DAPI) for 10 min. Between all incubation steps, the cells were washed three times for 5 min each with PBS containing 0.2% bovine serum albumin. Fluorescence signals were analyzed using an Olympus Fluoview 1000 confocal microscope (Olympus Corp, Tokyo, Japan). Three fields were analyzed for each of the samples.

### Cell viability assay

Cell viability was determined using the CCK-8 assay (Dojindo, Japan). According to the manufacturer’s instructions, cells were seeded in 96-well plates (10^4^ cells per well) in complete medium. The cells were then treated as indicated with drugs. CCK-8 reagent (10 μl) was added to each well, and 4 h later, each sample was measured at 450 and 630 nm.

### Cell death assay

Cell apoptosis was determined by the Annexin V Apoptosis Detection Kit APC (eBioscience Inc.) according to the manufacturer’s protocol. Approximately 2×10^5^ cells in each experimental group were washed with PBS and then resuspended. Annexin V (5 μl) and diluted PI (5 μl) were added to each sample. After 30 min, the cells were diluted with buffer and then analyzed by flow cytometry. Cell death in each group was assessed by counting cells showing fragmented nuclei after staining with DAPI or by trypan blue exclusion assay (>300 cells).

### Electron microscopy

Electron microscopy was conducted according to the standard procedures. Approximately 0.25-mm-thick SKM-1 cell slices were postfixed briefly in 2% osmium tetroxide in 0.1M cacodylate buffer, dehydrated in graded ethanol and embedded in epoxy resin. Ultrathin 60–70 nm sections were cut using a Leica ultra-microtome, mounted on 200-meshcopper grids. Grids were examined and photographed using a transmission electron microscope (Philips CM200).

### Transfection with small interfering RNAs (siRNAs)

SKM-1 cells were transfected with Atg3 small interfering RNA (siRNAs) using Lipofectamine 3000 transfection reagent (Invitrogen) according to manufacturer’s instructions. At 48 h post transfection, the knockdown efficiency at mRNA and protein level was determined by RT-PCR and Western immunoblotting analysis. Scrambled siRNA was used as negative control. siRNAs were supplied by Shanghai Gene Pharma Co.Ltd (Shanghai,China).

### Statistical analysis

All data were expressed as the mean ± standard deviation of three independent experiments. A one-way ANOVA with Tukey’s multiple comparison test was used to analyze the differences between groups. All statistical analyses were performed using SPSS 11.0 software. A p-value < 0.05 was considered statistically significant.

## Results

### 1. Downregulation of Atg3 in leukemia cells

**Fig 1:**
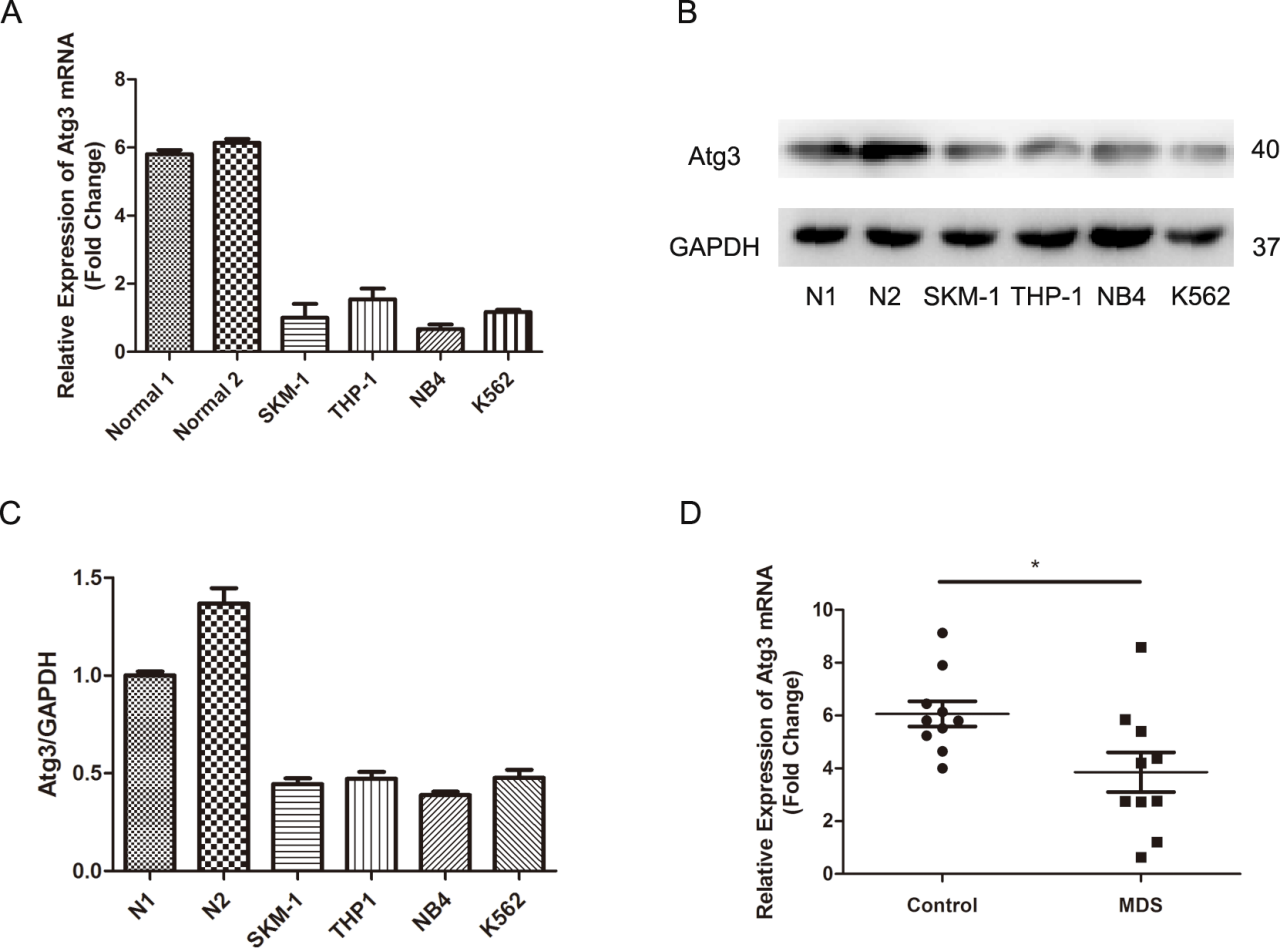
Analyses of Atg3 expression in leukemia cells. (A-C) Atg3 expression was analyzed by qRT-PCR and Western blotting in healthy bone marrow cells and four leukemia cell lines. Representative results from triplicate experiments are shown as the mean±SD. (D) Atg3 mRNA expression in healthy people (n = 10) and MDS patients (n = 10) was detected by qRT-PCR and plotted as mean ± SD of three independent experiments. *p<0.05.

To investigate the potential role of Atg3 in leukemia cells, we first analyzed the expression of Atg3 by real-time PCR and Western blotting in bone marrow cells of healthy people and human leukemia cell lines (SKM-1, THP-1, NB4, and K562). Both the Atg3 mRNA and protein expression were decreased in all four leukemia cell lines compared to healthy bone marrow cells (Fig 1A–1C). In addition, we detected the expression of Atg3 between healthy people (n = 10) and MDS patients (n = 10) by qRT-PCR (Fig 1D). Our results showed that Atg3 mRNA was significantly decreased in MDS patients’ bone marrow cells (MDS *versus* control: 6.063±0.475 *versus* 3.854±0.7469; p = 0.0225).

### 2. Lentivirus-mediated Atg3 overexpression in SKM-1 cells

**Fig 2:**
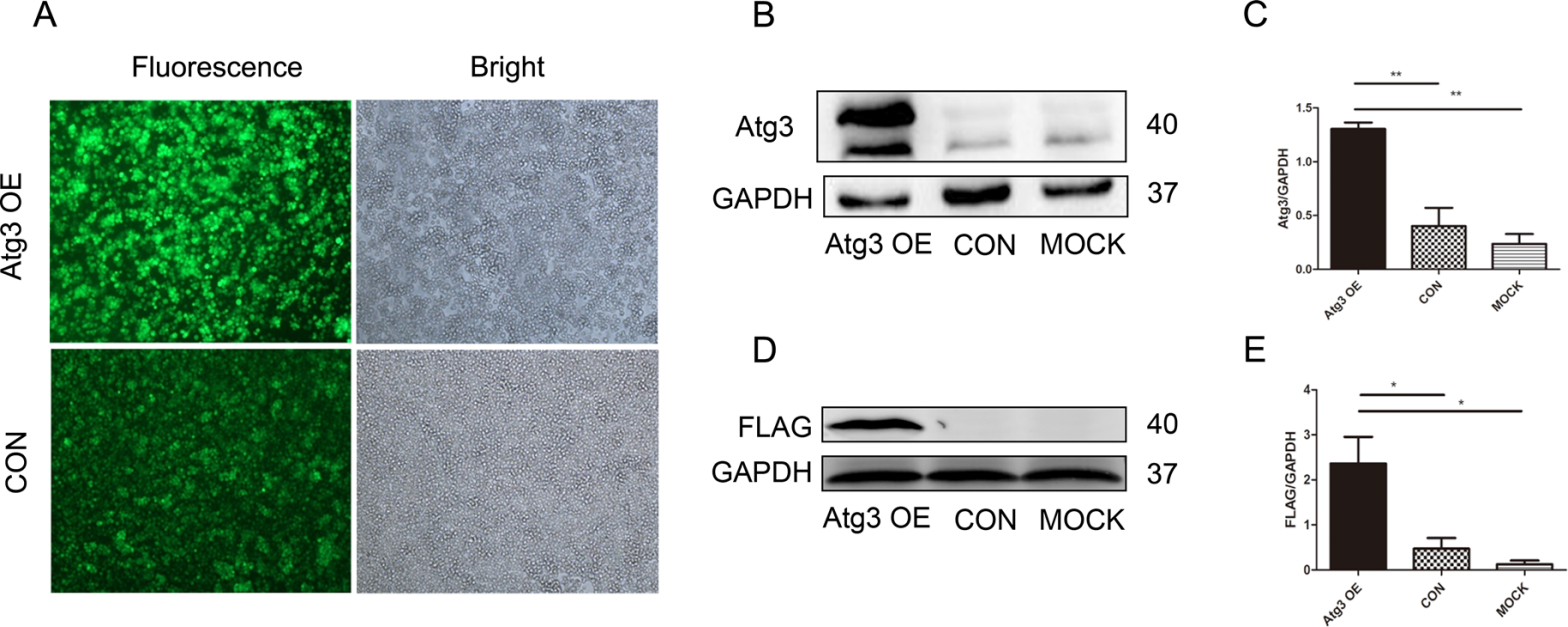
Lentivirus-mediated Atg3 overexpression in SKM-1 cells. (A) At 72 h post-transfection, SKM-1 cells transfected with FLAG-tagged ATG3-overexpressing vector and empty vector were detected by fluorescence and light microscopy. Western blotting of Atg3 protein (40 kD band) in SKM-1 cells detected by Atg3 (B and C) and FLAG (D and E) antibodies. Representative results from triplicate experiments are shown as the mean±SD. *p<0.05, **p<0.01.

To explore the function of the Atg3 protein, SKM-1 cells were transfected with a FLAG-tagged ATG3-overexpressing vector or an empty vector lentivirus. At 72 h after transfection, GFP expression was examined using fluorescence microscopy. The transfection efficiency of each group was above 80% (Fig 2A). The protein expression was further confirmed by Western blotting. The level of the Atg3 protein was significantly greater in the Atg3 overexpression group (Atg3 OE group) than the control group and mock group (Fig 2B and 2C, Fig 2D and 2E).

### 3. Atg3 in SKM-1 cells induces AKT-mTOR dependent autophagy

**Fig 3:**
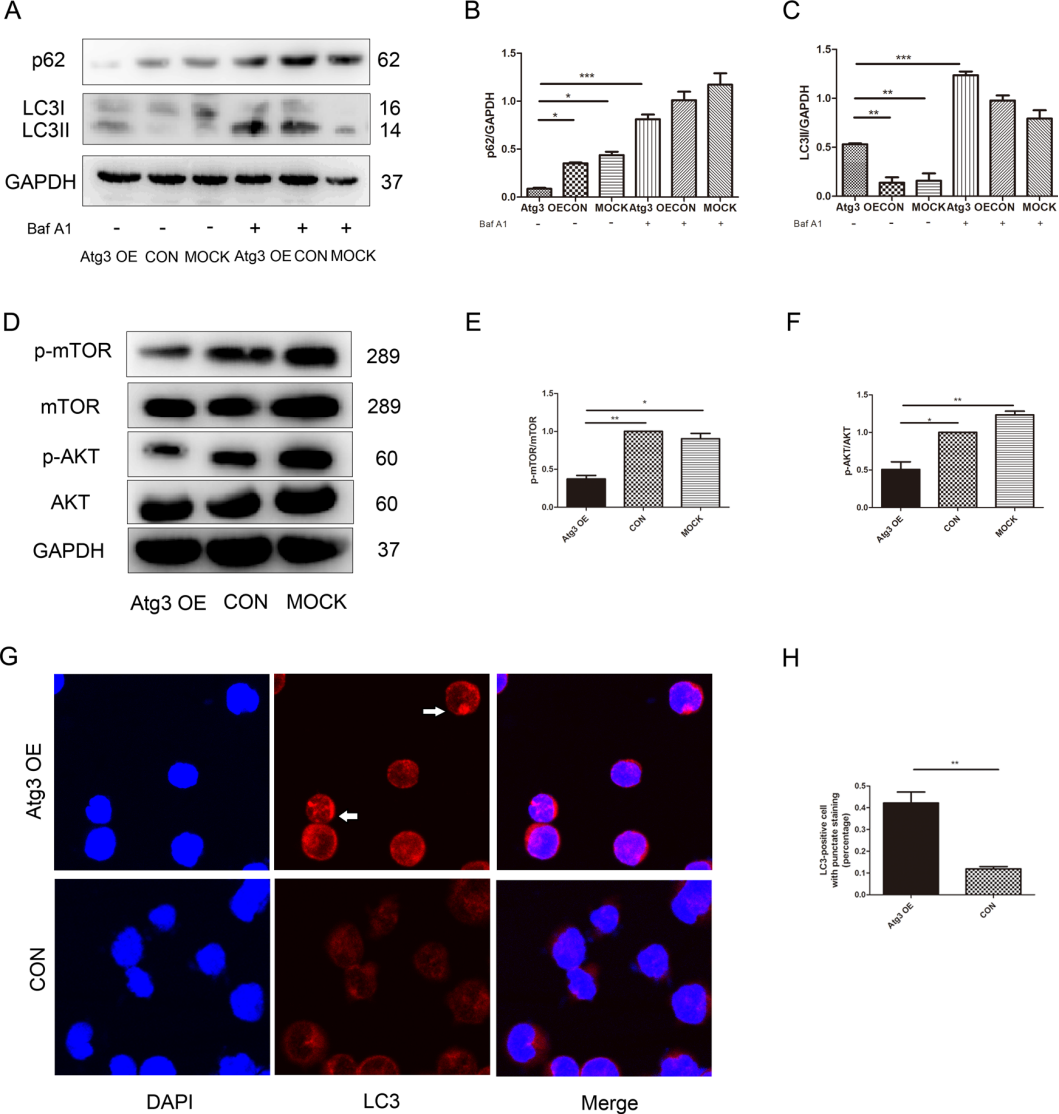
Atg3 overexpression in SKM-1 cells induces autophagy. (A-C) At 96 h post-transfection, cells of each group were subjected to Western blotting for LC3 and p62 after Baf A1 treatment for 2 h.(D-F) The expression of proteins involved AKT, pAKT, mTOR and p-mTOR were analyzed at 96 h post-transfection. GAPDH served as a loading control. Protein levels were normalized to GAPDH expression. (G and H) The formation of LC3 puncta was assayed by confocal microscopy, and the percentage of cells with punctate staining was calculated. Representative results from triplicate experiments are shown as the mean±SD (n = 3). *p<0.05, **p<0.01, ***p<0.001.

To investigate whether Atg3 is a direct activator of autophagic flux, we detected LC3 conversion by Western blotting. LC3 is widely used to monitor autophagy, and the amount of LC3-II correlates with the number of autophagosomes. Atg3 overexpression increased the expression of LC3-II in SKM-1 cells (Fig 3A and 3C). Sequestosome 1 (p62) is a long-lived scaffolding protein involved in the transport of ubiquitinated proteins destined for proteasomal digestion. Targets of the p62 protein are incorporated into the autophagosome and serve as a selective substrate of autophagy. Atg3 overexpression decreased p62 levels (Fig 3A and 3B). In order to observe the effect of Atg3 on autophagic flux further, we treated cells with 100nM Baf A1 that inhibited lysosomal acidification and blocked proteolytic digestion for 2 h. The expression of LC3-II and p62 in the Atg3 OE group was increased significantly after Baf A1 treatment, which indicated that Atg3 activated autophagic flux (Fig 3A–3C). These results were further confirmed by confocal microscopy. The formation of endogenous LC3 puncta was increased after Atg3 overexpression. In addition, the LC3 expression revealed a transition from a diffuse cytoplasmic pattern to a punctate membrane pattern, suggesting the localization of LC3 to the autophagosome (Fig 3G and 3H). To further investigate the molecular and cellular consequences resulting from Atg3 overexpression, we examined the AKT-mTOR signaling pathway using Western blotting. Compared to the control group and mock group, the Atg3 OE group showed decreased levels of p-AKT and p-mTOR (Fig 3D–3F). These data suggest that Atg3 might regulate autophagy through an AKT-mTOR signaling pathway.

### 4. Atg3 overexpression-induced cell death is caspase-independent

**Fig 4:**
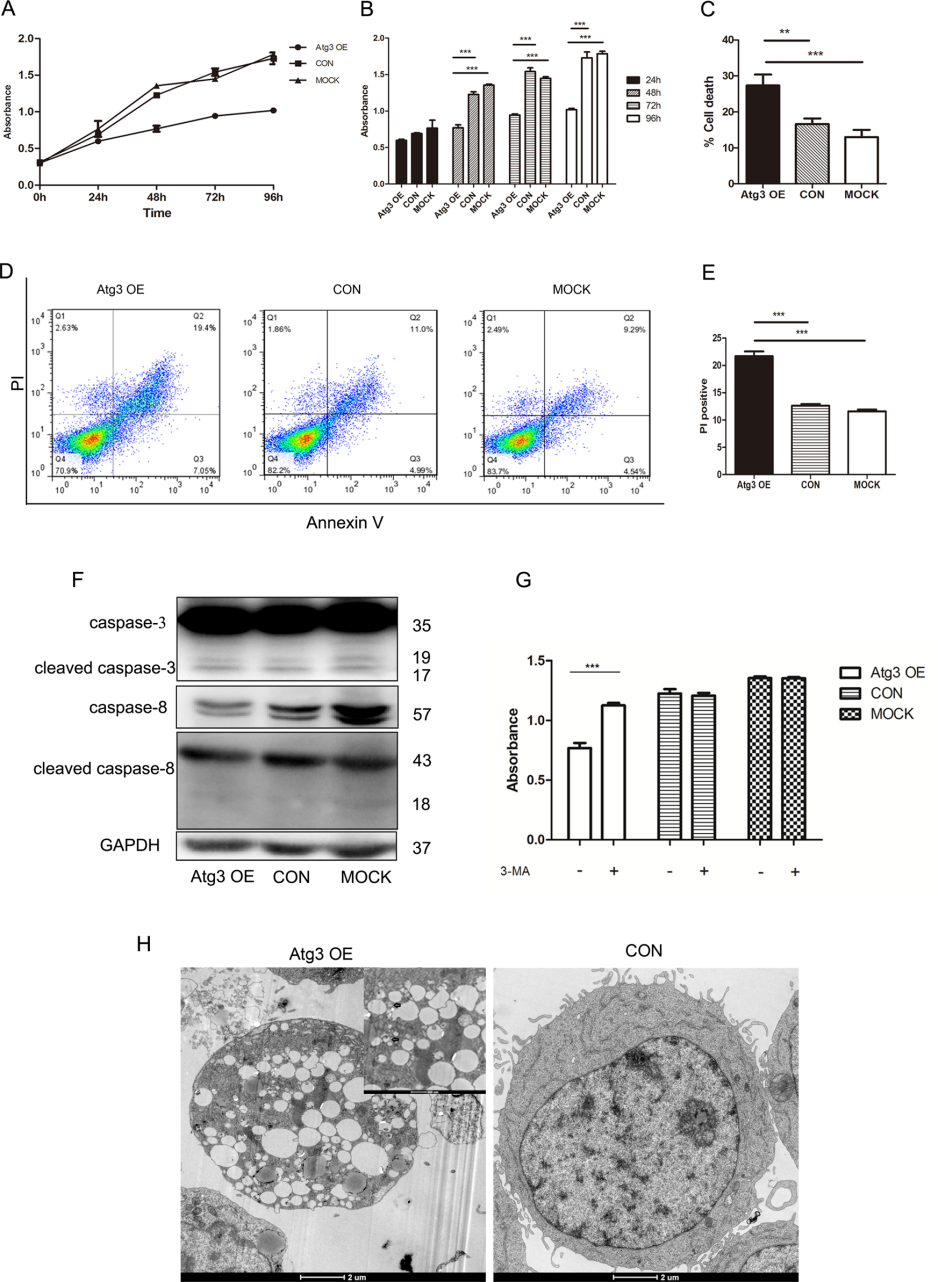
Effects of Atg3 on the cell viability and cell death of SKM-1 cells. (A and B) Plots of growth curves over time as indicated for cells of each group. (C) Analyses of cell death for the Atg3 OE group, control group and mock group using the trypan blue exclusion assay. (D) Representative histograms of Annexin V-APC/PI double staining flow cytometry. (E) Cell death indicated by the PI positive rate. (F) Western blotting of caspase-3 and caspase-8 expression in cells of each group. (G) Analyses of cell viability in the absence or presence of 2 mM 3-MA for 48 h. (H) Ultrastructural changes of each group detected by electron microscopy. The arrows indicate autophagosomes. Experiments were repeated at least three times, and representative results are shown as the mean±SD. ***p<0.001.

To investigate whether the autophagy induced by Atg3 has an effect on cell growth of SKM-1 cells, the growth curves of SKM-1 cells were assessed by CCK-8 assay following lentivirus-mediated overexpression of Atg3. Compared to the control group and mock group, cells of the Atg3 OE group showed reduced cell growth, especially at 48 h, 72 h and 96 h (Fig 4A and 4B). Then, we evaluated cell death by trypan blue staining. The percentages of cell death for the Atg3 OE group, control group and mock group were 27.33±1.76, 16.67±0.88, 13±1.16, respectively (Fig 4C). Compared to the control group and mock group, the Atg3 OE group showed increased cell death. We further detected cell death using Annexin V/ PI dual staining flow cytometry (Fig 4D). Our data showed that the PI positive rates of the Atg3 OE group, control group and mock group were 21.68±1.53, 12.62±0.54, 11.59±0.53, respectively (Fig 4E). However, we did not observe activation of the caspase signaling pathway, including caspase-3 and caspase-8 (Fig 4F). To further explore the type of cell death, we added the autophagy inhibitor 3-MA to each group. 3-MA is an autophagy inhibitor that has been reported to inhibit the activity of PI3 kinase and block the formation of autophagosomes. We incubated cells of each group in the absence or presence of 2 mM 3-MA for 48 h, and the percentage of cell death in the Atg3 OE group was significantly reduced (Fig 4G). These results suggest that Atg3 overexpression-induced cell death is autophagic cell death rather than caspase-dependent cell death. We also observed the ultrastructural changes of each group using electron microscopy. Compared to the control group and mock group, cells of the Atg3 OE group showed a large lipid droplet and autophagosomes with double membrane bound structures (Fig 4H).

### 5. SKM-1 cells overexpressing Atg3 are hypersensitive to bortezomib treatment

**Fig 5:**
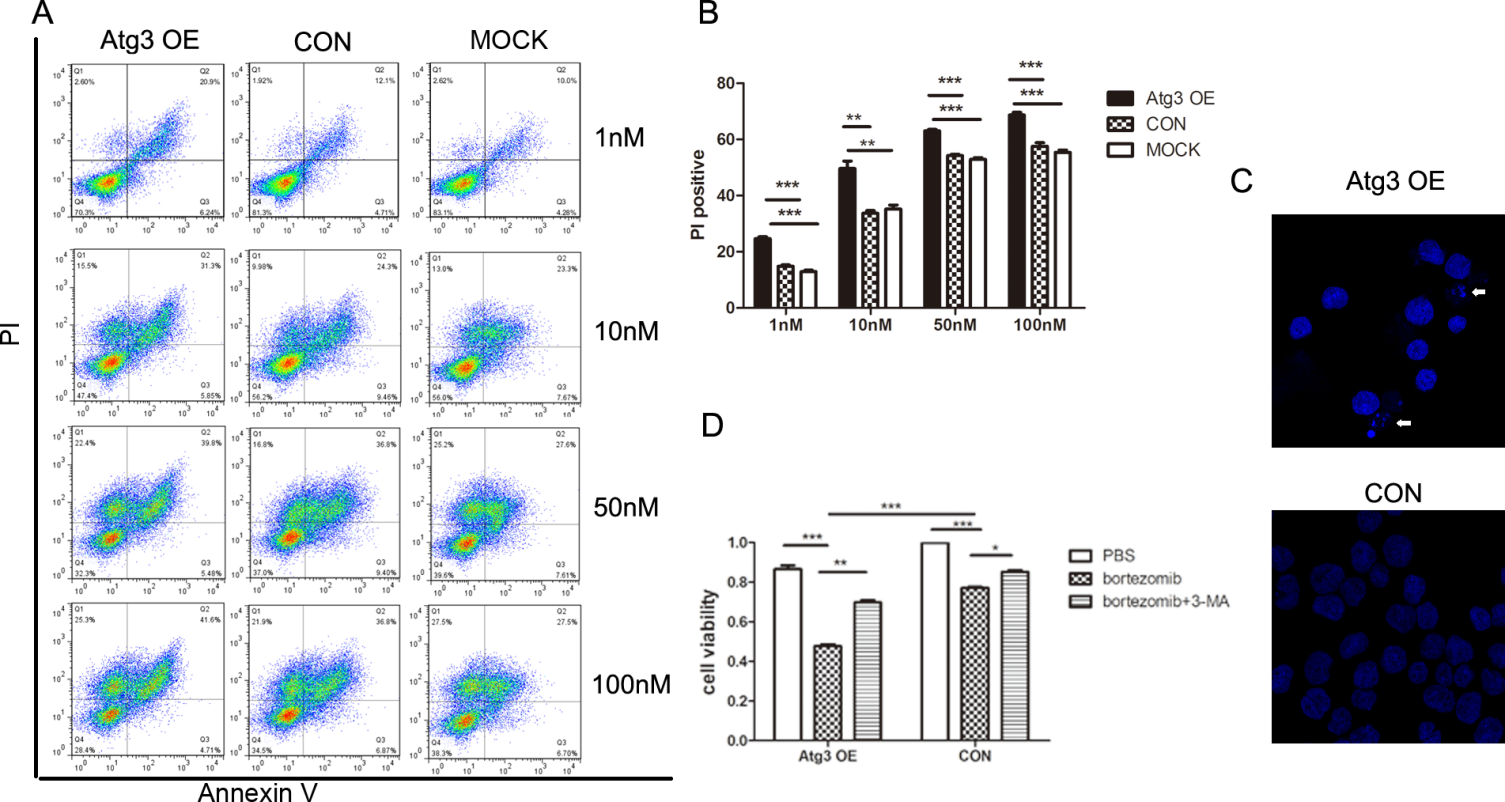
Upregulation of autophagy by Atg3 overexpression increases the sensitivity of SKM-1 cells to bortezomib treatment. (A) Annexin V-APC/ PI double staining flow cytometry of each group treated with increasing concentrations of bortezomib (1 nM, 10 nM, 50 nM, and 100 nM). (B) The PI positive rate of each group with the indicated concentration of bortezomib. (C) Cellular nuclei were stained by DAPI and visualized using fluorescence microscopy at 24 h after 10 nM bortezomib treatment. (D) Cell viability of each group was analyzed by CCK-8 assay in the absence or presence of 10 nM bortezomib for 24 h. All data are representative of three independent experiments and are shown as the mean±SD. *p<0.05, **p<0.01, ***p<0.001.

Considering that autophagy is a double-edged sword for cancer cells, especially in the condition of stress, we investigated the effect of Atg3 following bortezomib treatment. First, we treated three groups of cells with different concentrations of bortezomib (1, 10, 50, and 100 nM) for 24 h. The PI positive rate of each group was dose-dependent. Compared to the control group and mock group, the Atg3 OE group had a statistically significant increased cell death rate (Fig 5A and 5B). Then, we treated cells of each group with bortezomib at a concentration of 10 nM. At 24 h following treatment, cells of the Atg3 OE group showed nucleolus pyknosis and nuclear fragmentation (Fig 5C). Similarly, cell growth was significantly inhibited in the Atg3 OE group 24 h following bortezomib treatment (Fig 5D). These findings suggest that Atg3 significantly enhances bortezomib-mediated cytotoxicity in SKM-1 cells.

### 6. Autophagy is required for bortezomib-induced cytotoxicity

**Fig 6:**
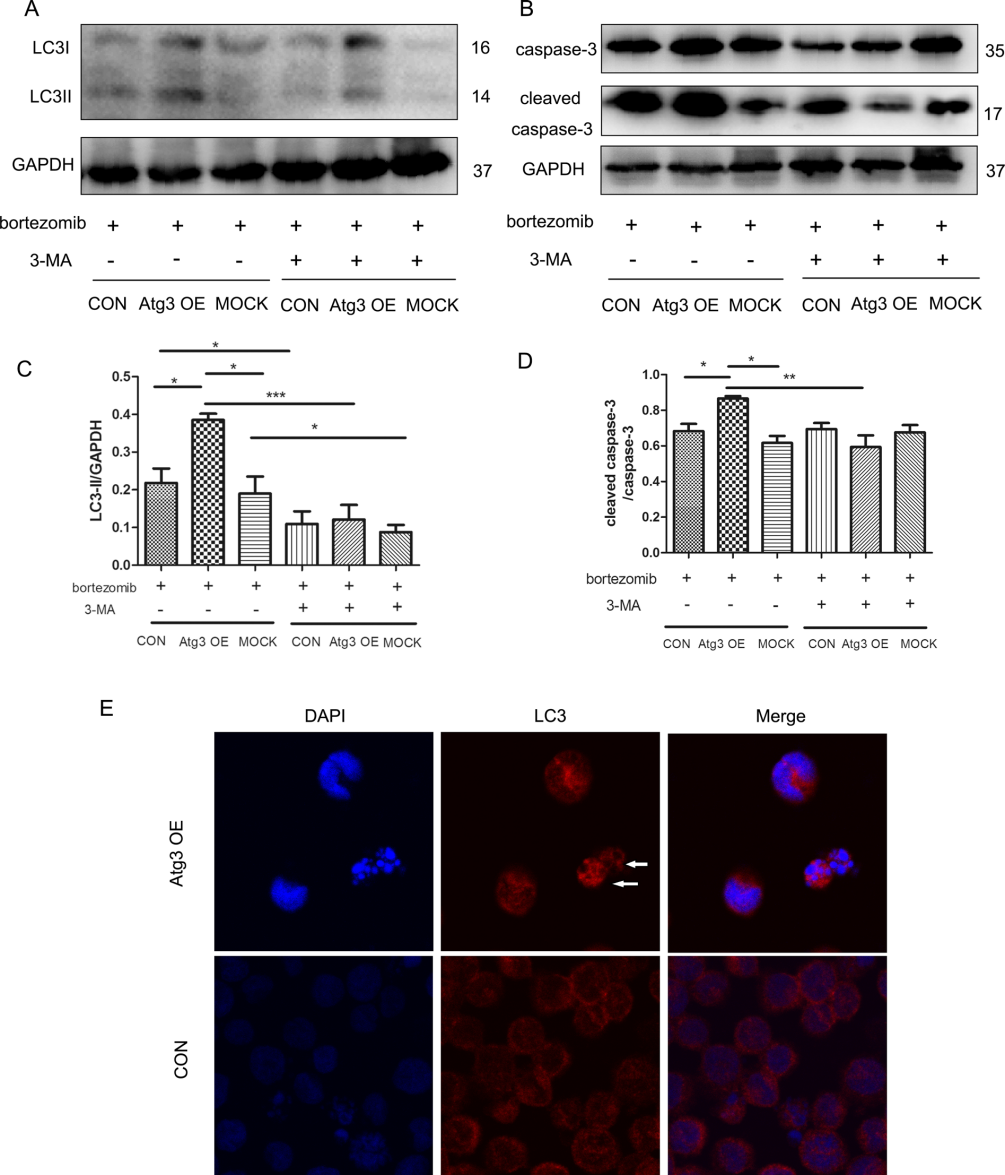
Autophagy is required for bortezomib-induced cytotoxicity. (A and C) Cells of each group were treated with bortezomib (10 nM) for 12 h. LC3 was analyzed by Western blotting in the presence or absence of 3-MA (2 mM) 4 h before the end of bortezomib treatment. (B and D) Caspase-3 and cleaved caspase-3 were detected by Western blotting. GAPDH was used as a loading control. At least three individual experiments were detected by Western blotting and representative results are shown as the mean±SD. (E) At 12 h after bortezomib (10 nM) treatment, the formation of LC3 puncta was assayed by confocal microscopy before the end of bortezomib treatment. Immunofluorescence experiments were repeated at least three times and representative results are shown.

**Fig 7:**
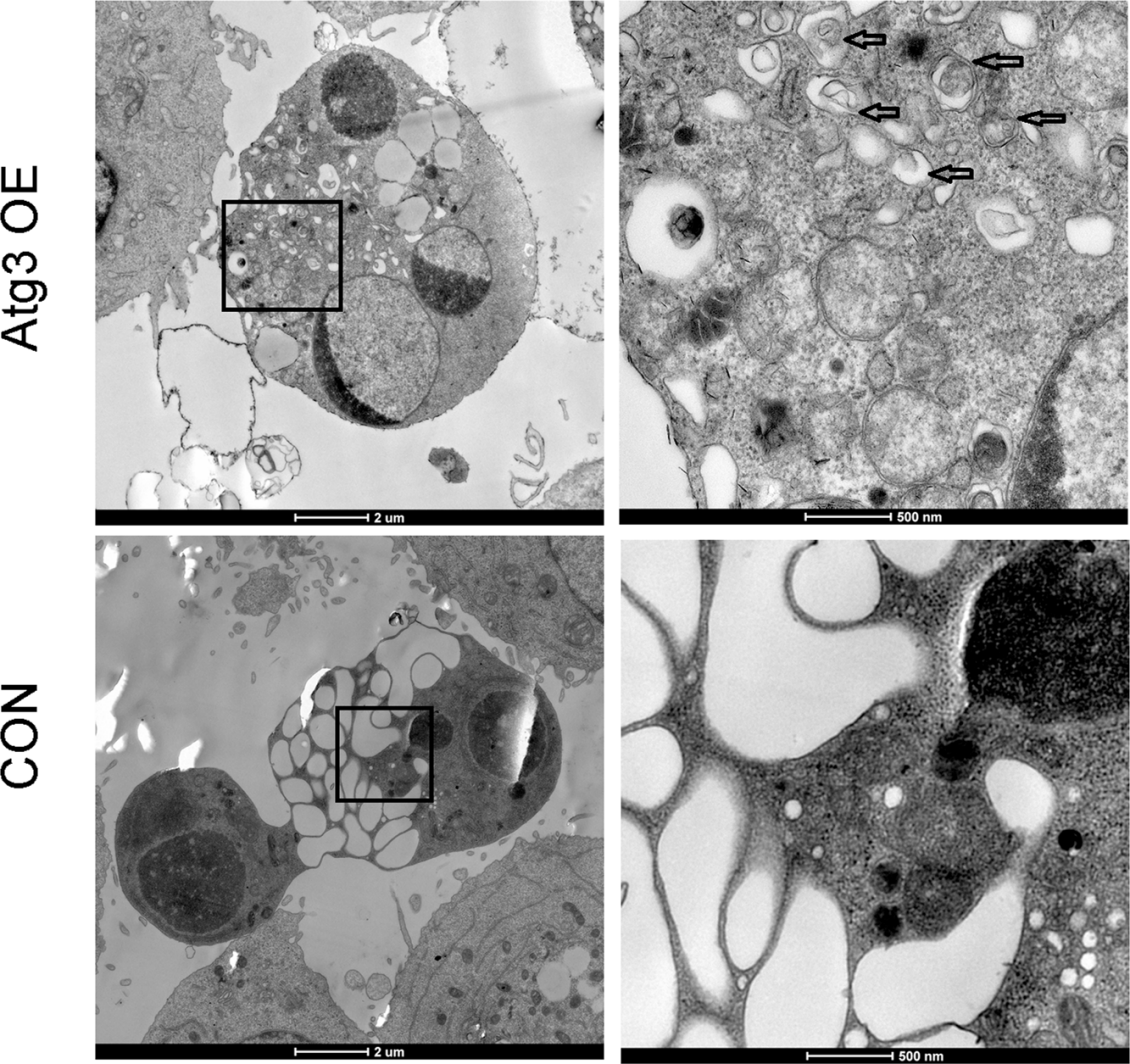
Cells of each group were treated with 10 nM of bortezomib for 12 h and then subjected to scanning electron microscopy. All data are representative of three independent experiments. *p<0.05, **p<0.01, ***p<0.001.

**Fig 8:**
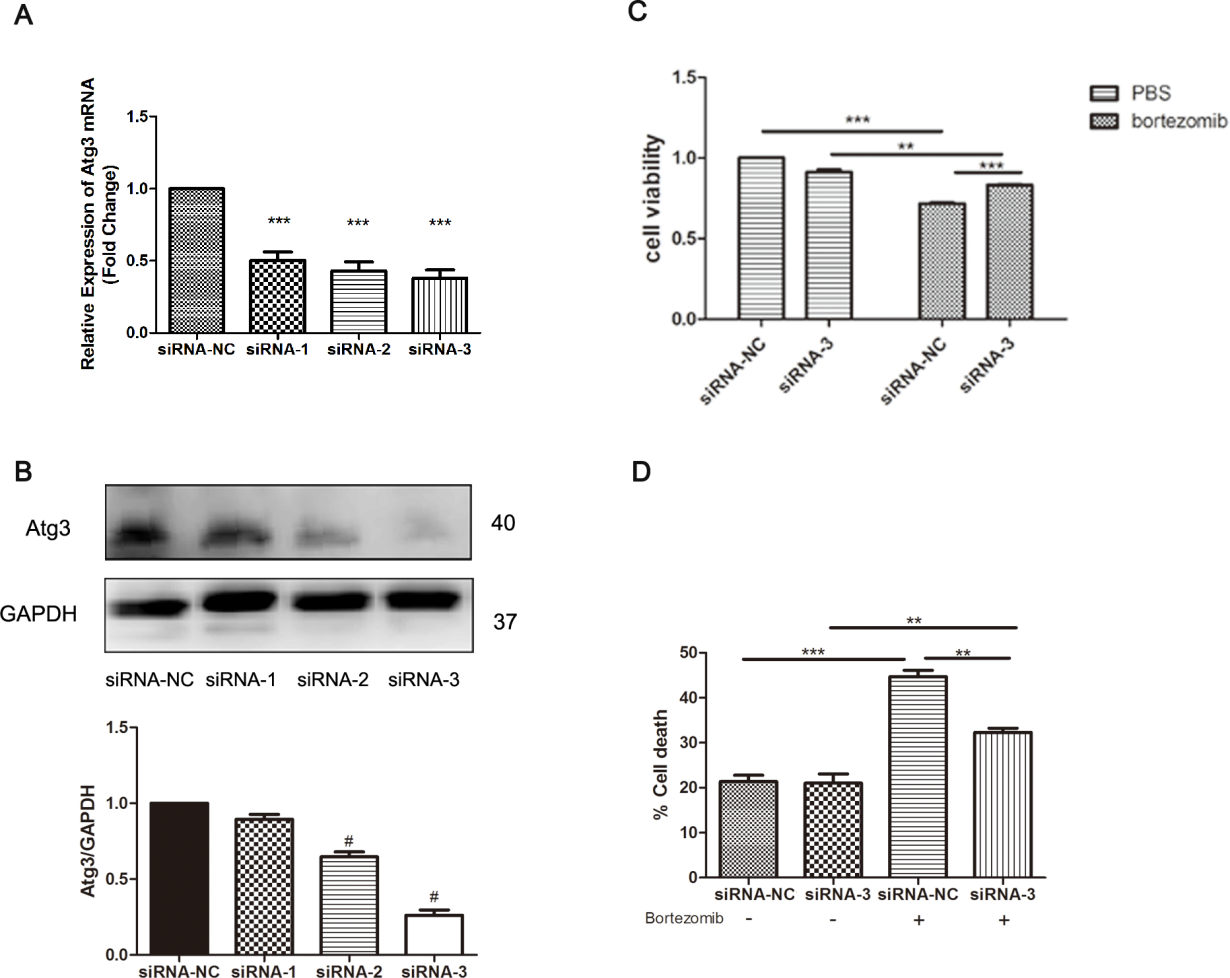
(A-B) Knock down of Atg3 mRNA and protein by siRNA was confirmed by qRT-PCR and Western blotting analysis. SKM-1 cells were treated with PBS or 10 nM bortezomib for 24 h in combination with Atg3 siRNA or NC siRNA. (C) Cell growth of SKM-1 cells was assessed by CCK-8 assay. (D) Cell death was analyzed by trypan blue exclusion assay. All data are representative of three independent experiments and are shown as the mean±SD. #p<0.05, **p<0.01, ***p<0.001.

To investigate the role of autophagy mediated by Atg3 overexpression after bortezomib treatment, 3-MA (2 mM) was applied for 4 h before the end of bortezomib treatment. As expected, autophagy was activated after bortezomib treatment for 12 h with increased LC3II expression in the Atg3 OE group. After applying 2 mM 3-MA, autophagy was inhibited (Fig 6A and 6C). To further investigate the effects of autophagy induced by Atg3 overexpression on cell apoptosis and chemosensitivity, caspase-3 and cleaved caspase-3 were analyzed by Western blotting. Compared to the control group and mock group, there was an increase in cleaved caspase-3 in the Atg3 OE group. Interestingly, after applying 3-MA, apoptosis was also decreased as a result of decreased levels of cleaved caspase-3 (Fig 6B and 6D). We observed the characteristics of cell death and nuclear fragmentation in the Atg3 OE group and control group after bortezomib treatment. Moreover, in the Atg3 OE group, the nuclear fragments were surrounded by LC3 puncta (Fig 6E). Cell viability was increased as a result of bortezomib and 3-MA treatment for 24 h (Fig 5D). Electron microscopy studies showed severe autophagy in dead cells with nuclear fragmentation of Atg3 OE group (Fig 7). To further evaluate the role of Atg3 in the bortezomib treatment, siRNAs of Atg3 were transduced into SKM-1 cells and siRNA-3 could reduce the expression of Atg3 in both mRNA and protein levels (Fig 8A and 8B). Then SKM-1 cells were treated with PBS or 10 nM bortezomib in combination with Atg3 siRNA or NC siRNA. Atg3 siRNA-3 blocked the cell growth inhibition induced by bortezomib (Fig 8C). Besides, cells transfected with Atg3 siRNA-3 had a decreased cell death rate compared with cells transfected with siRNA- NC under the treatment of bortezomib (Fig 8D). Taken together, these data show that Atg3 induced autophagy is required for bortezomib-induced cytotoxicity.

## Discussion

The acquisition of cytogenetic abnormalities in MDS plays a vital role in the progression of this disease. However, the exact molecular mechanisms of this process remain largely unknown. In the current study, we systematically evaluated the effect of Atg3 overexpression in the SKM-1 cell line. Our results showed that Atg3 induced autophagy and caspase-independent cell death. Moreover, Atg3 regulated chemotherapy sensitivity by inducing autophagic cell death and increasing apoptosis. Inhibition of Atg3 overexpression-induced autophagy by a pharmacological autophagy inhibitor reduced the chemotherapy sensitivity of bortezomib. To the best of our knowledge, the present study is the first to report the role of Atg3 in bortezomib treatment.

Atg3, which is involved in autophagosome formation, is a key protein in autophagy signaling [19]. The Atg3 protein is composed of three characteristic regions: a E2 core region, a flexible region, and a handle region, which are necessary for the interaction with Atg7 and Atg8 [20]. Previously, we showed that Atg3 may play a role in the transformation of MDS [5,21]. The current study showed that Atg3 was downregulated in leukemia cell lines compared to healthy bone marrow cells. To further evaluate whether Atg3 plays a functional role in MDS/AML, we assessed the changes of biological characteristics in SKM-1 cells after overexpression of Atg3. We found that Atg3 overexpression induced autophagy in SKM-1 cells, manifested by increased LC3II levels and decreased p62 expression. Moreover, Atg3 overexpression induced caspase-independent cell death, which can be overcome by the autophagy inhibitor 3-MA. Numerous studies have showed that the AKT-mTOR signaling pathway plays a central role in the induction of autophagy [22–24]. Consistent with these studies, our data showed that Atg3 overexpression inhibited the AKT-mTOR signaling pathway. This result suggests that Atg3 could induce autophagy through the AKT-mTOR signaling pathway.

The UPS is a type of protein degradation pathway that maintains cell viability through the selective turnover of targeted proteins [25,26]. In recent years, numerous reports have demonstrated that active crosstalk exists between the UPS and the autophagy pathway [27–29]. A recent study revealed that HACE1, a ubiquitin ligase, could accelerate autophagic flux by ubiquitinating OPTN and promoting its interaction with p62/SQSTM1 to form the autophagy receptor complex [30]. Moreover, LC3, which plays an essential role in autophagy, can be degraded by the 20S proteasome in an ATP- and ubiquitin-independent manner [31]. The present study showed that autophagy contributed to the cytotoxic effects of bortezomib. Consistent with these previous studies, our study suggests the potential linkage between autophagy and the UPS.

Bortezomib is a potent and selective proteasome inhibitor that efficiently affects the UPS. Bortezomib has been shown to activate autophagy in various diseases. However, autophagy plays different roles in different diseases. Recent studies have showed that bortezomib provokes cell-protective autophagy, which shields tumor cells and promotes drug resistance [32–34]. On the contrary, other evidence has shown that bortezomib exerts its anti-tumor effects by inducing autophagic cell death [14]. These results can be explained by the fact that autophagy favors or counteracts cell death signaling depending on the cellular context, the cell line and the different modes of autophagy induction. However, elucidating the role of autophagy may not only strengthen our understanding of tumorigenesis but also provide new insight into the treatment of tumors. Our study showed that after the bortezomib treatment in the Atg3 OE group, autophagy was also activated. In addition, the autophagy inhibitor can partially decrease the cell death rate induced by bortezomib. These results showed that Atg3 overexpression induced autophagy-mediated cell death after bortezomib treatment. Autophagy is described as a double-edged sword, and there is much controversy about the role of autophagy in cell survival and death, especially for cancer cells [35,36]. Our findings are consistent with studies indicating that autophagy acts as a tumor-suppressing mechanism.

Autophagy and apoptosis are two different determinants of cell fate under physiologic and pathologic conditions. Cell death is most commonly associated with apoptosis, known as type I programmed cell death (PCD). As a different type of PCD from apoptosis, increasing evidence has demonstrated that autophagy-mediated cell death plays a vital role in tumor development and therapy [36,37]. Autophagy and apoptosis have a complex relationship with each other. Recently, connections and crosstalk between apoptosis and autophagy have been under investigation. It has been reported that Atg5 contributes to autophagic cell death by interacting with Fas-associated protein with death domain (FADD) [38]. Autophagic cell death requires the genes ATG7 and beclin1 and is induced by caspase-8 inhibition [39]. To investigate the relationship between autophagy and apoptosis, we examined the levels of activated caspase-3. After bortezomib treatment, there were increased levels of cleaved caspase-3 in the Atg3 OE group. These results may be caused by other functions of Atg3 in apoptosis or by some downstream cellular signaling pathways. Further elucidation of this phenomenon is currently under study.

In conclusion, the findings of the present study implicate Atg3 as a potential target to attenuate the progression of MDS/AML and for therapeutic interventions in MDS.

## Supporting Information

S1 Table Supporting Information. Sequence of specific siRNAs for *ATG3* and negative control.
(DOCX)

S2 Table Supporting Information. Clinical characteristics of MDS patients.
(DOCX)
