# Supplementary material for: Atg3 Overexpression Enhances Bortezomib-Induced Cell Death in SKM-1 Cell
Source: PLoS One. 2016 Jul 8;11(7):e0158761. doi: 10.1371/journal.pone.0158761 (PMC4938461; doi:10.1371/journal.pone.0158761)
Supplement: S1 Table — Sequence of specific siRNAs for ATG3 and negative control. (DOCX) [file pone.0158761.s001.docx]

**Table 1 Sequence of specific siRNAs for *ATG3* and negative control**

|  | sense : | antisense: |
| --- | --- | --- |
| ATG3-homo-686 | 5'GGUGCAAACAGAUGGAAUATT 3' | 5'UAUUCCAUCUGUUUGCACCTT 3' |
| ATG3-homo-895 | 5'GCUGCAGAUAUGGAAGAAUTT 3' | 5'AUUCUUCCAUAUCUGCAGCTT 3' |
| ATG3-homo-1278 | 5'GGGAGAACUUGGAGUUCAUTT 3' | 5'AUGAACUCCAAGUUCUCCCTT 3' |
| Negative control | 5'UUCUCCGAACGUGUCACGUTT3' | 5'ACGUGACACGUUCGGAGAATT3' |
