# Supplementary material for: Atg3 Overexpression Enhances Bortezomib-Induced Cell Death in SKM-1 Cell
Source: PLoS One. 2016 Jul 8;11(7):e0158761. doi: 10.1371/journal.pone.0158761 (PMC4938461; doi:10.1371/journal.pone.0158761)
Supplement: S2 Table — Clinical characteristics of MDS patients. (DOCX) [file pone.0158761.s002.docx]

**Table 2 Clinical characteristics of MDS patients**

| Patient no. | Sex | Age | WHO  classification |
| --- | --- | --- | --- |
| 1 | male | 80 | RA |
| 2 | male | 45 | RAEB-2 |
| 3 | female | 60 | MDS/sAML |
| 4 | male | 75 | RAEB-1 |
| 5 | male | 61 | RAEB-2 |
| 6 | female | 52 | RAEB-2 |
| 7 | male | 68 | RAEB-2 |
| 8 | female | 45 | RAEB-1 |
| 9 | female | 71 | RAEB-1 |
| 10 | male | 49 | RCMD |
